# Three‐dimensional printers applied for the production of beam blocks in total body irradiation treatment

**DOI:** 10.1002/acm2.13592

**Published:** 2022-03-15

**Authors:** Manuel Maerz, Marius Treutwein, Jan Nabo, Barbara Dobler

**Affiliations:** ^1^ Department of Radiotherapy Regensburg University Medical Center Regensburg Germany; ^2^ Department for Mathematics and Computer Science Ostbayerische Technische Hochschule Regensburg Regensburg Germany

**Keywords:** 3D printer, electron beam apertures, photon beam blocks, total body irradiation

## Abstract

**Purpose:**

Total body irradiation (TBI) in extended source surface distance (SSD) is a common treatment technique before hematopoietic stem cell transplant. The lungs are organs at risk, which often are treated with a lower dose than the whole body.

**Methods:**

This can be achieved by the application of blocks. Three‐dimensional (3D) printers are a modern tool to be used in the production process of these blocks.

**Results:**

We demonstrate the applicability of a specific printer and printing material, describe the process, and evaluate the accuracy of the product.

**Conclusion:**

The blocks and apertures were found to be applicable in clinical routine.

## 1 INTRODUCTION

The introduction of multileaf collimators for radiotherapy linear accelerators (linacs) started more than 30 years ago. This type of collimator reduced the need for photon blocks compared to the standard rectangle jaw collimator. The blocks served to reduce the dose to the normal tissue and to the organs at risk. The increase in application of intensity modulated techniques has superseded the production of photon blocks. The dose to the organs at risk is reduced in the planning process by application of appropriate dose volume objectives. However, for special applications photon blocks are still useful. Lung shields to avoid pneumonitis after total body irradiation (TBI) are rather common.

Although for TBI treatments intensity modulating techniques have been developed^1^, ^2^, ^3^, ^4^, ^5^, ^6^ photon blocks are still applied in many centers, using different techniques at extended source surface distance (SSD). Examples published in the past 15 years are of sweeping beam technique^7^, ^8^, ^9^ and translational couch treatment.^10^, ^11^ Additionally, conventional fixed beam techniques with standing or lying patients are widespread as well.^12^, ^13^, ^14^, ^15^ The principles of these techniques are well established and have been described decades ago.^16^, ^17^

We searched for a contemporary solution for the production of photon blocks and electron apertures concerning the age of our former block cutting device and the related software. The software had been released for Windows 7 only and the support for Windows 7 ended in January 2020.^18^ Some publications demonstrated that three‐dimensional (3D) printers can be used for the production of electron apertures.^19^, ^20^

Dedicated foam cutting devices have not only been much more expensive than a low‐cost 3D printer. Additionally, as Michiels et al. pointed out,^19^ they require storage place, especially for the bulky foam panels that cause costs as well. Michiels et al. demonstrated equivalent dose distributions with an electron aperture produced by a conventional mold of polystyrene (PS) foam compared to a 3D printed one. 3D printers can avoid common errors which occur with PS cutting devices and PS molds such as rounding of edges by cutting too fast, extended kerf by using to high temperature of the cutting wire, and deformation of the mold by the hot block alloy.

However, a systematic review about the application of 3D printers in radiation oncology by Rooney et al. demonstrated no work about the production of photon blocks.^21^ For TBI treatments a specific dose to the lungs has to be applied. For this purpose the lung blocks are used as partial shield and the transmission is determined by the height of the blocks. Therefore, the height must be regarded as a critical parameter, which is uncritical for electron apertures. An extended SSD requires additional geometric corrections compared to standard geometry where the patient is treated on the treatment table.

We decided to develop a procedure for the production of photon blocks for TBI using a commercial 3D printer. This printer was also used for the production of electron apertures to boost the partially shielded regions of the thoracic wall. The TBI technique has been described in the following section in short.

## 2 MATERIAL AND METHODS

We apply a TBI technique, which has been described by Härtl et al.^8^ This sweeping beam technique uses an extended SSD. The patient lies on a low couch. The top of the couch is 117.5 cm below the isocenter. The sagittal plane of the body coincides with the rotation plane of the gantry. A plate of 10 mm Makrolon® polycarbonate is positioned 33 cm above the couch top to provide full skin dose.^22^ Lung shields are arranged on this plate.

The thoracic wall in the shielded region is boosted by electron fields as recommended by van Dyk et al.^23^ because the bone marrow of the ribs belongs to the target volume. These electron fields are applied in standard geometry with the patient on the couch of the linear accelerator at a SSD of 110 cm.

We use a Canon Aquilion TSX‐201A scanner (Canon Medical Systems Corporation, Tochigi, Japan) to generate a whole body computed tomography (CT) scan of the patient in supine position. This is used for contouring and dose calculations as described by Härtl et al.^8^ A scan of the thorax region only serves for contouring of the lungs in prone position. Both scans apply helical scanning with slice distance 1.0 cm.

**FIGURE 1:**
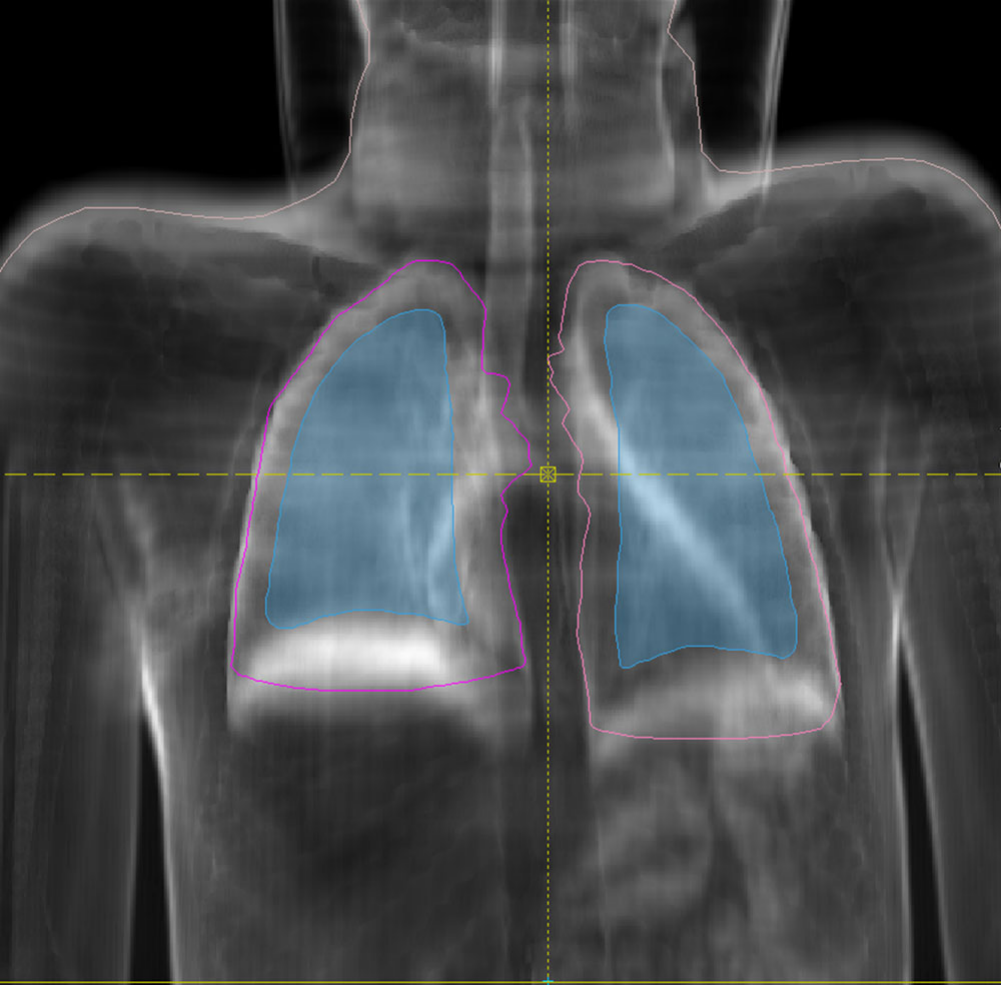
Beam's eye view for gantry angle of 350° of the thoracic region in a lung window. The lung blocks are shown in bright blue, the lung contours in pink and purple, and the yellow cross hair marks the central beam

The configuration of the blocks is performed using the treatment planning system (TPS) Oncentra (Nucletron, an Elekta company, Veenendal, Netherlands). The lung blocks are configured in the beam's eye view of a fixed beam. The gantry angle is 350° so that the central axis is at about the middle of the longitudinal lung extension (Figure 1). The lung blocks are applied to reduce the dose to the center of the lungs to 7.0 Gy.^8^ The shape of the blocks is manually adapted by the radiotherapist, considering the diaphragm and a margin of about 1 cm to the thoracic wall. The divergence of the blocks is calculated for the gantry angle of 350°. However, during the treatment the gantry rotates. Thus, the dose gradient is blurred in the patient's longitudinal direction as presented by Hautmann et al.^24^

The radiation therapy (RT) plan DICOM file is exported to a program in Matlab® code (V. R2020b, The MathWorks, Inc., Natick, Massachusetts, US). This program is an in‐house developed product, which generates a 3D model of the divergent blocks under consideration of the geometric parameters like source to skin distance , block tray distance, block height, and others. For this purpose, the contours of the blocks are projected to several positions between the bottom and top layer of the blocks and a voxelized binary 3D model of the molds is created. The block height is calculated as described by Härtl et al.^8^ Then the 3D model is converted to a 3D surface definition. The molds for electron aperture cutouts are calculated directly as complementary shapes of the photon blocks regarding the different geometric setup, implying that the dimensions of the blocks for the low couch had to be converted to the dimensions of the cutouts in the electron absorbers applied for the patient on the linac treatment table according the intercept theorem.

We used a 3D printer of type Prusa i3 MK3S (Prusa Research, Prag, Czech Republic). This printer had the lowest cost of purchase in a comparison of six 3D printers with comparable potential for clinical utility according to Chen et al.^25^ It is a very common 3D printer because of its manufacturing and price.^26^ The Prusa i3 is an open source project and the latest version MK3S was released in 2017.^27^

The surface file from the Matlab program is imported in the PrusaSlicer software (V. 2.2.0, open source) which creates a gcode file for the control of the printer. The printer runs with most commercial printing materials. We chose a generic polyethylene terephthalate modified with glycol (PETG) with a diameter of 1.75 mm (Renkforce, Conrad Electronic SE, Hirschau, Germany). The printing temperature of this material is 230 – 270°C and corresponds to the window between melting and boiling range.^28^ A bed temperature of 60 – 80°C is recommended. Terpenning gives the Vicat softening temperature of PETG with 181°F (82.8°C).^29^ This parameter describes a specified point of softening when the material is exposed to an elevated temperature.

**FIGURE 2:**
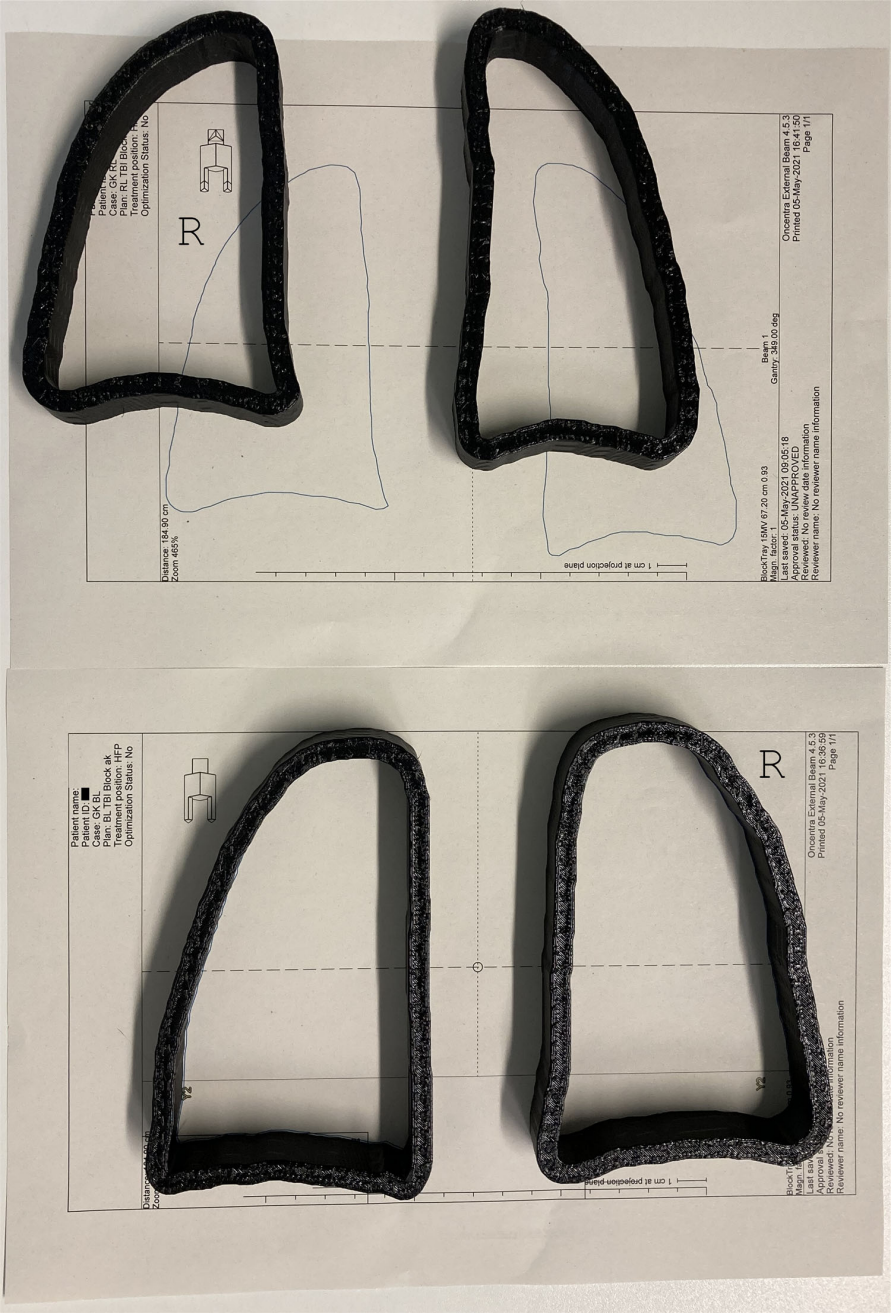
Photon block molds for both lungs of one patient. Top for supine and bottom for prone positioning on paper sheets from the treatment planning system (TPS). The letter “R” indicates the right side

Figure 2 shows the molds for the photon blocks of one patient on sheets printed from the TPS. The sheets allow a direct comparison of the planned and the 3D printed form. The molds were filled with MCP96 alloy to build the photon blocks. This is the same block material as it has been used in the former production process with molds cut from PS foam. MCP96 is a low melting alloy with a melting point of 96°C and a density of 9.85 g/cm^3^. Its main ingredient is lead. The thermostat of the melting pot is set to about 120°C. In the present investigation, we included patients getting a total dose of 12 Gy only. Then, the specified dose to the central lung of 7 Gy is achieved by a block height of 20 ‐ 32 mm according to our experience.

**FIGURE 3:**
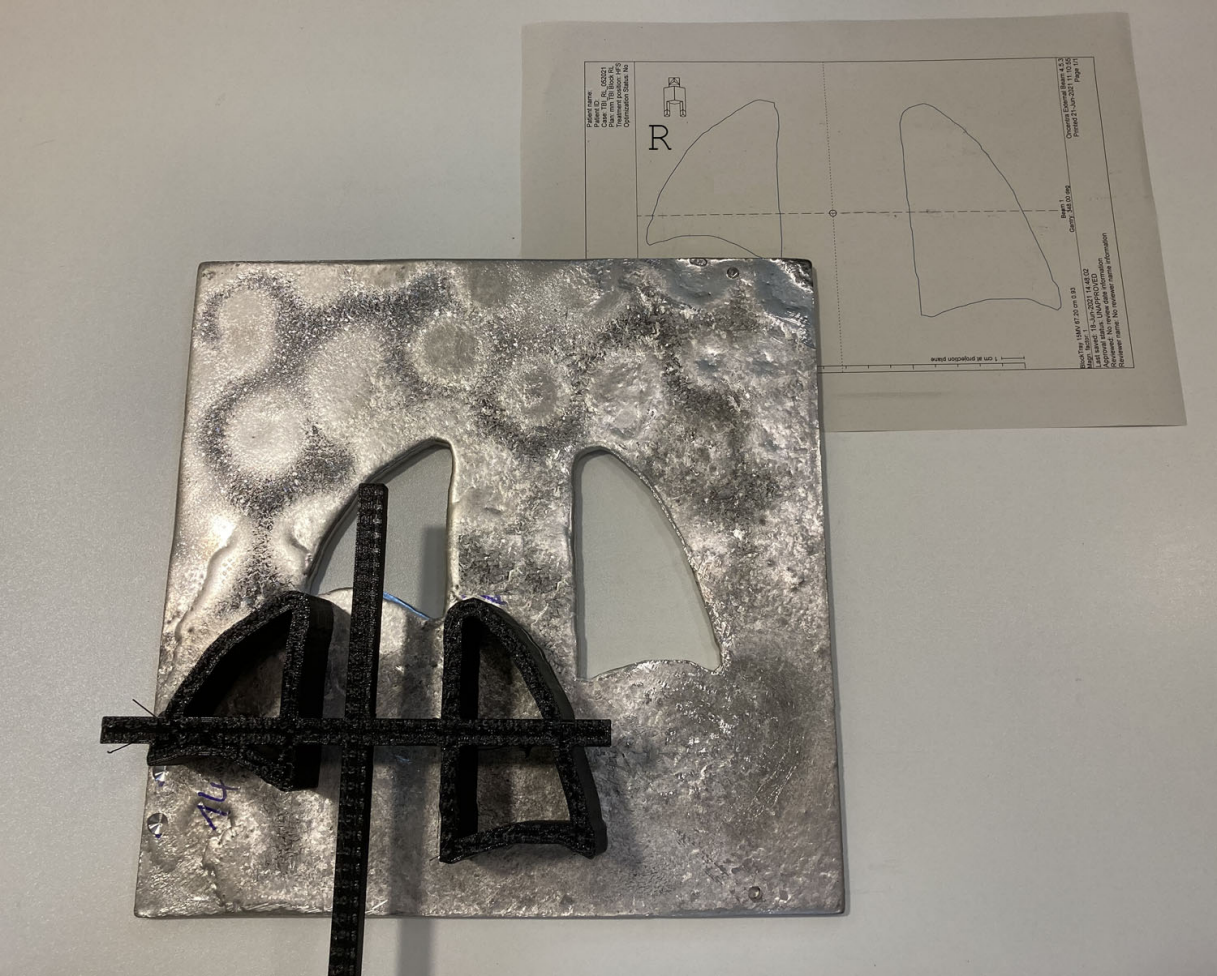
Electron aperture with three‐dimensional (3D) printed shape and paper sheet from the treatment planning system (TPS) for supine positioning. The letter “R” on the sheet indicates the right side

Figure 3 shows the mold for an electron aperture. The cross at the top serves to keep the two cutouts in the correct position to each other and within the outer frame. For electron apertures, the outer frame (not in the image) is filled with liquid MCP alloy surrounding the mold. The height of the outer frame, which is 10 mm, defines the thickness of electron apertures. The outer contour of the electron mold has to correspond to the inner contour of the photon block molds in the projection on the patient's skin. Figures 2 and 3 are of different patients.

The printer is equipped with a print nozzle of 0.6 mm in diameter. The layer's height was set to 0.35 mm with two external contours. As infill of the object, a gyroid pattern with a density of 15% was used with three layers of floor and four layers of roof. Support structures for overhanging parts were generated.

The molds, blocks, and apertures for three consecutive patients have been included in this investigation. They consisted of two photon lung blocks and one electron aperture for supine and prone positions, each.

## 3 RESULTS

The geometric shape of the molds for photon blocks corresponds very well to the requirements (Table 1). Arrangement of the molds on a printout from the TPS is a fast quality check (Figure 2). The divergence of the blocks is of minor importance for the applied technique. First, due to the extended source to block distance and the flat height the influence of the divergence is small and second, as described in the previous section, the rotating gantry blurs the block's shadow. Nevertheless, we perform a regular quality assurance of the divergence of photon block molds twice a year to ensure a standard shape. The inclination of the sidewalls is correct within 0.5°, which corresponds to the measurement accuracy. Therefore, measures of the footprint and height only were controlled quantitatively using a vernier caliper.

**TABLE 1:** Measures in millimeters of photon blocks in the plan, molds, ready block, and difference of block and plan values

		**Planned dimension of block in mm**			**Measured dimension of mold in mm**			**Measured dimension of block in mm**			**Difference of measured and planned dimension of block in mm**		
**Patient ID**	**Positioning**	**x Plan**	**y Plan**	**z Plan**	**x Mold**	**y Mold**	**z Mold**	**x Block**	**y Block**	**z Block**	**Δ x Block‐plan**	**Δ y Block‐plan**	**Δ z Block‐plan**
1	Supine right	59.4	111.3	21.0	60.0	110.9	21.4	60.0	111.8	22.1	0.6	0.5	1.1
1	Supine left	51.5	132	21.0	51.3	131.2	21.4	52.2	132.2	22.2	0.7	0.2	1.2
1	Prone left	68.8	133.5	23.0	68.1	132.2	23.4	69.2	133.4	24.2	0.4	‐0.1	1.2
1	Prone right	72.5	130.9	23.0	72.6	130.5	23.5	73.0	131.8	24.4	0.5	0.9	1.4
2	Supine right	60.1	117.5	30.0	59.6	116.6	30.5	60.6	117.6	31.2	0.5	0.1	1.2
2	Supine left	68.2	143.3	30.0	67.9	142.5	30.3	68.6	143.6	31.2	0.4	0.3	1.2
2	Prone left	63.9	156.3	32.0	64.2	156.5	32.2	64.6	157.0	32.8	0.7	0.7	0.8
2	Prone right	62.0	135.8	32.0	61.8	135.4	32.2	62.3	135.6	32.8	0.3	‐0.2	0.8
3	Supine right	59.7	82.5	21.0	59.6	81.8	21.2	60.3	82.2	22.8	0.6	‐0.3	1.8
3	Supine left	52.8	107.4	21.0	52.2	106.1	21.2	54.0	107.6	22.0	1.2	0.2	1.0
3	Prone left	54.5	121.2	20.0	53.8	120.3	20.3	55.3	120.9	21.2	0.8	‐0.3	1.2
3	Prone right	66.4	87.4	20.0	65.6	86.4	20.4	67.2	85.5	20.2	0.8	‐1.9	0.2
										Mean	0.6	0.0	1.1
										Standard deviation	0.2	0.7	0.4

The deformation of the sidewalls has been controlled visually. Even higher samples showed only air gaps of submillimeter width between the sidewall and a ruler attached in top–bottom direction.

**FIGURE 4:**
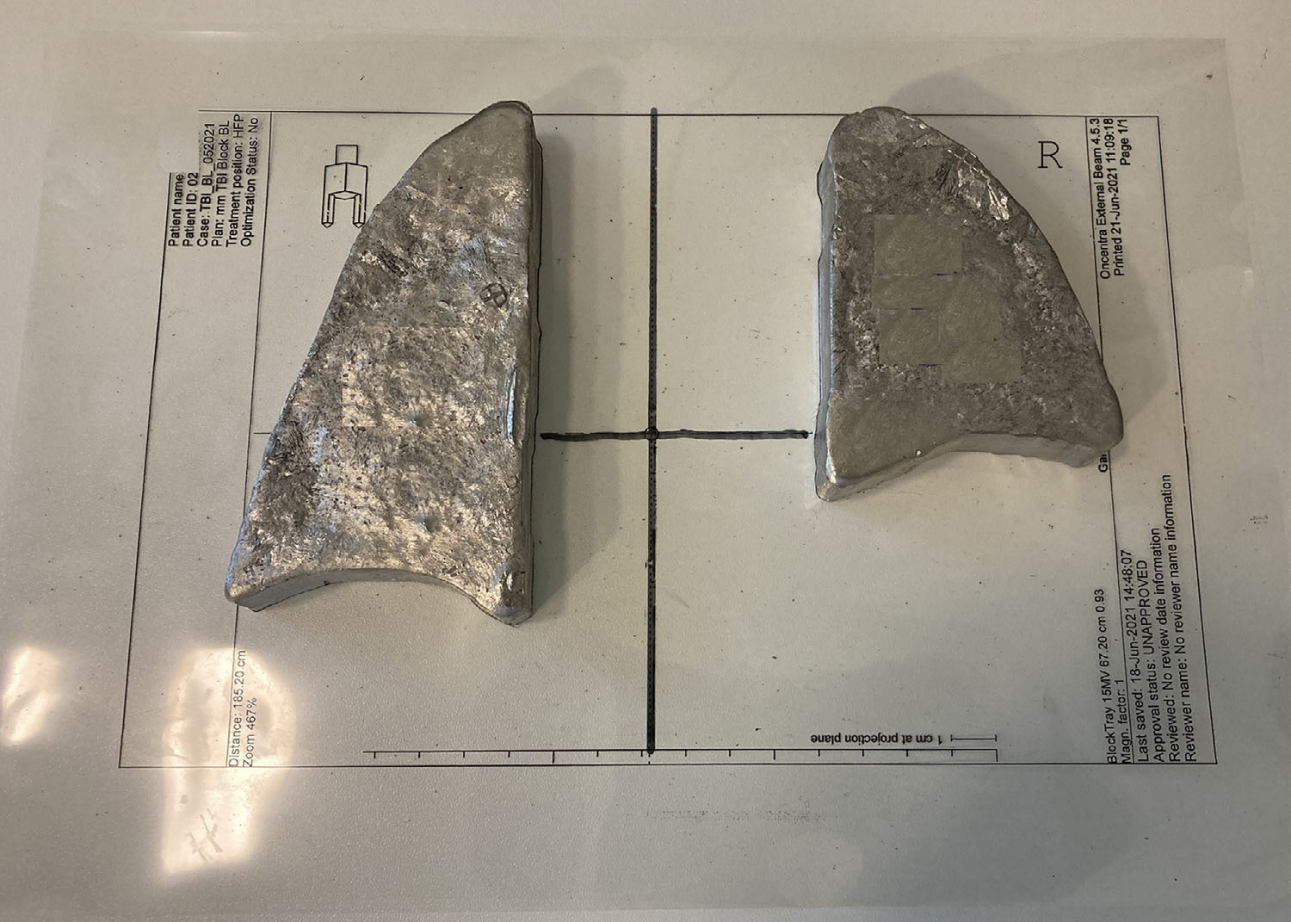
Photon blocks on a transparency to facilitate the arrangement above the patient for prone positioning. The letter “R” indicates the right side

The average of linear measures in x and y direction deviated only some tenths of a millimeter from the values of the TPS. The blocks were slightly larger, but in most cases deviated less than 1 mm from the planning measure. The planned height of photon blocks should be achieved by printing the mold in the specified height (Table 1, column “z plan”). The height of the molds was always larger than intended—up to 0.5 mm. The height of the blocks was again increased up to 0.8 – 1.8 mm above the target value. For the electron apertures, only a visual check on the printouts from the TPS has been performed and showed similar agreement as the photon blocks. Figures 3 and 4 demonstrate examples of photon blocks and electron apertures, respectively. The average print time for one patient was 16.2 hours.

## 4 DISCUSSION

The material of the actual medical product – the blocks and apertures – is unchanged compared with the former production process with PS foam cutting devices. Therefore, there is no need for control other than the geometric measures of the products. The blocks can be handled in the accustomed manner.

The accuracy and reproducibility of 3D printers was investigated by George et al.^30^ They found it better than 1 mm and in most cases better than 0.5 mm, even in the print layer dimension which they regarded as the most inaccurate. In our production process the print layer thickness affects the height of the block molds and therefore the divergence and thickness of the blocks. The dimensions of our printing products showed a similar precision. The precision was suitable for clinical cases as the block dimensions were only marginally deviating from the target size.

PETG demonstrated sufficient heat resistance when filled with molten MCP96 without visible deformation. The upper surface of the blocks and apertures shows some irregularities and less smoothness than the sidewalls in submillimeter range, which results from slight temperature gradients during the cooling process. This is similar to the former production process with molds of PS foam. If a higher precision for the height of the blocks is needed than was achieved by the founding process, a milling machine or manual or machine‐assisted sanding can be applied.

The x‐ and y‐dimensions of our blocks were slightly larger than the measured dimensions of the molds (Table 1). The mold walls might have yielded when being filled with the alloy, because the Vicat softening temperature was below the temperature of the liquid MCP96 alloy.

We have explained why we did not consider the divergence of the photon blocks in detail. However, our regular quality assurance shows no deviation in the modelling of the divergent sidewalls. Therefore, this production process should also be applicable for TBI treatments with fixed beam, where the correct divergence is more important. Similarly, it is possible for other large field treatments such as total nodal irradiation,^31^ malignant pleural mesothelioma,^32^ Hodgkin's lymphoma in female pediatric patients,^33^ or when blocks are preferred, for example, in developing countries.^34^

The 3D printing process takes more time than cutting a mold from PS foam. This must be considered in the treatment preparation. The printing time could be reduced by using two printers simultaneously. Moreover, the second printer can serve as a backup if the first printer fails. At our hospital the time to prepare for TBI treatments is 3 weeks or more, having about 20 patients per year. From March to December we had produced molds for eight individual patients with our 3D printer. Thus, the printing time is not a critical factor. Until now, no breakdown of a printer during the printing process has been observed. Therefore, most prints can run overnight.

3D printers are a promising tool in the production of absorbing blocks and apertures. They offer additional useful applications such as printing of bolus material, quality assurance phantoms, or brachytherapy applicators.^21^ 3D printers can thus reduce or eventually eliminate the need of purchasing commercial medical products.^35^, ^36^

## AUTHOR CONTRIBUTIONS

Manuel Maerz participated in the development of the project, developed the program code, set up the production process, and contributed details to the manuscript; Marius Treutwein initiated the project and participated in its development, evaluated the results, and drafted the manuscript; Jan Nabo performed first investigations in material and methods and created test molds and blocks; Barbara Dobler participated in the development of the project, supervised it, and helped to draft the manuscript. All authors read the final version and agreed to it.

## CONFLICT OF INTEREST

The authors declare that there is no conflict of interest.
